# Supplementary material for: Ripply3 overdosage induces mid-face shortening through Tbx1 downregulation in Down syndrome models
Source: PLoS Genet. 2025 Sep 22;21(9):e1011873. doi: 10.1371/journal.pgen.1011873 (PMC12469710; doi:10.1371/journal.pgen.1011873)
Supplement: S1 Table — (DOCX) [file pgen.1011873.s001.docx]

| Landmarks Cranium | |
| --- | --- |
| 1 | Nasale: Intersection of nasal bones, rostral point |
| 2 | Nasion: Intersection of nasal bones, caudal point |
| 3 | Bregma: intersection of frontal bones and parietal bones at midline |
| 4 | Intersection of parietal bones with anterior aspect of interparietal bone at midline |
| 5 | Intersection of interparietal bones with squamous portion of occipital bone at midline |
| 6 | Opisthion, midsagittal point on the posterior margin of the foramen magnum |
| 7 | Center of alveolar ridge over maxillary incisor, right side |
| 8 | Anterior Intersection of frontal process of maxilla with frontal bone, right side. |
| 9 | Anterior notch on frontal process lateral to infraorbital fissure, right side |
| 10 | Intersection of frontal process of maxilla with frontal and lacrimal bones, right side |
| 11 | Frontal-squasmosal intersection at temporal crest, right side |
| 12 | Intersection of zygoma (jugal) with zygomatic process of temporal, superior aspect, left side |
| 13 | Intersection of zygoma (jugal) with zygomatic process of temporal, inferior aspect, left side |
| 14 | Most posteroinferior point on the superior portion of the tympanic ring, right side |
| 15 | Center of alveolar ridge over maxillary incisor, left side |
| 16 | Anterior Intersection of frontal process of maxilla with frontal bone, left side. |
| 17 | Anterior notch on frontal process lateral to infraorbital fissure, left side |
| 18 | Intersection of frontal process of maxilla with frontal and lacrimal bones, left side |
| 19 | Frontal-squasmosal intersection at temporal crest, left side |
| 20 | Intersection of zygoma (jugal) with zygomatic process of temporal, superior aspect, right side |
| 21 | Intersection of zygoma (jugal) with zygomatic process of temporal, inferior aspect, right side |
| 22 | Most poteroinferior point on the superior portion of the tympanic ring, left side |
| 23 | Most anterior point of the anterior palatine foramen, right side |
| 24 | Most posterior point of the anterior palatine foramen, right side |
| 25 | Most infero lateral point on premaxilla-maxilla suture, right side |
| 26 | The anterior most point on the central ant/post axis of the right molar alveolus |
| 27 | Intersection of zygomatic process of maxilla with zygoma (jugal), inferior surface, right side |
| 28 | Lateral intersection of maxilla and palatine bone posterior to the third molar, right side |
| 29 | Joining of squasmosal body to zygomatic process of squasmosal, right side |
| 30 | Most inferior aspect of posterior tip of medial pterygoid process, right side |
| 31 | Most anterior point of the anterior palatine foramen, left side |
| 32 | Most posterior point of the anterior palatine foramen, left side |
| 33 | Most infero lateral point on premaxilla-maxilla suture, left side |
| 34 | The anterio most point on the central ant/post axis of the left molar alveolus |
| 35 | Intersection of zygomatic process of maxilla with zygoma (jugal), inferior surface, left side |
| 36 | Lateral intersection of maxilla and palatine bone posterior to the third molar, left side |
| 37 | Joining of squasmosal body to zygomatic process of squasmosal, left side |
| 38 | Most inferior aspect of posterior tip of medial pterygoid process, left side |
| 39 | Basion, midsagittal point on the anterior margin of the foramen magnum |

**Table S1:** 39 Cranium Landmarks for morphometric analysis.
