# Supplementary material for: Ripply3 overdosage induces mid-face shortening through Tbx1 downregulation in Down syndrome models
Source: PLoS Genet. 2025 Sep 22;21(9):e1011873. doi: 10.1371/journal.pgen.1011873 (PMC12469710; doi:10.1371/journal.pgen.1011873)
Supplement: S2 Table — (DOCX) [file pgen.1011873.s002.docx]

| Landmarks Mandible | |
| --- | --- |
| 1 | Apex of coronoid process, Right side |
| 2 | Intersection of molar alveolar rim and base of coronoid process, Right side |
| 3 | Anterior edge of alveolar process where first molar hits alveolus at the midline, Right side |
| 4 | Superior-most point on incisor alveolar rim at midline (at bone-tooth junctions), Right side |
| 5 | Inferior-most point on incisor alveolar rim at midline (at bone-tooth junction), Right side |
| 6 | Inferior point on mandibular symphysis, Right side |
| 7 | Anterior edge of the coalescence of curve of masseteric ridge with post-symphyseal rugged area, Right side |
| 8 | Tip of mandibular angle, Right side |
| 9 | Posterior midline point on condyle, Right side |
| 10 | Anterior midline point on condyle, Right side |
| 11 | Anterior edge of the mental foramen, Right side |
| 12 | Apex of the coronoid process, left side |
| 13 | Intersection of molar alveolar rim and base of coronoid process, left side |
| 14 | Anterior edge of alveolar process where first molar hits alveolus at the midline, left side |
| 15 | Superior-most point on incisor alveolar rim at midline (at bone-tooth junctions), left side |
| 16 | Inferior-most point on incisor alveolar rim at midline (at bone-tooth junction), left side |
| 17 | Inferior point on mandibular symphysis, left side |
| 18 | Anterior edge of the coalescence of curve of masseteric ridge with post-symphyseal rugged area, left side |
| 19 | Tip of mandibular angle, left side |
| 20 | Posterior midline point on condyle, left side |
| 21 | Anterior midline point on condyle, left side |
| 22 | Anterior edge of the mental foramen, left side |

**Table S2:** 22 Mandible Landmarks for Morphometric Analysis.
