# Supplementary figures and images for: Ripply3 overdosage induces mid-face shortening through Tbx1 downregulation in Down syndrome models
Source: PLoS Genet. 2025 Sep 22;21(9):e1011873. doi: 10.1371/journal.pgen.1011873 (PMC12469710; doi:10.1371/journal.pgen.1011873)

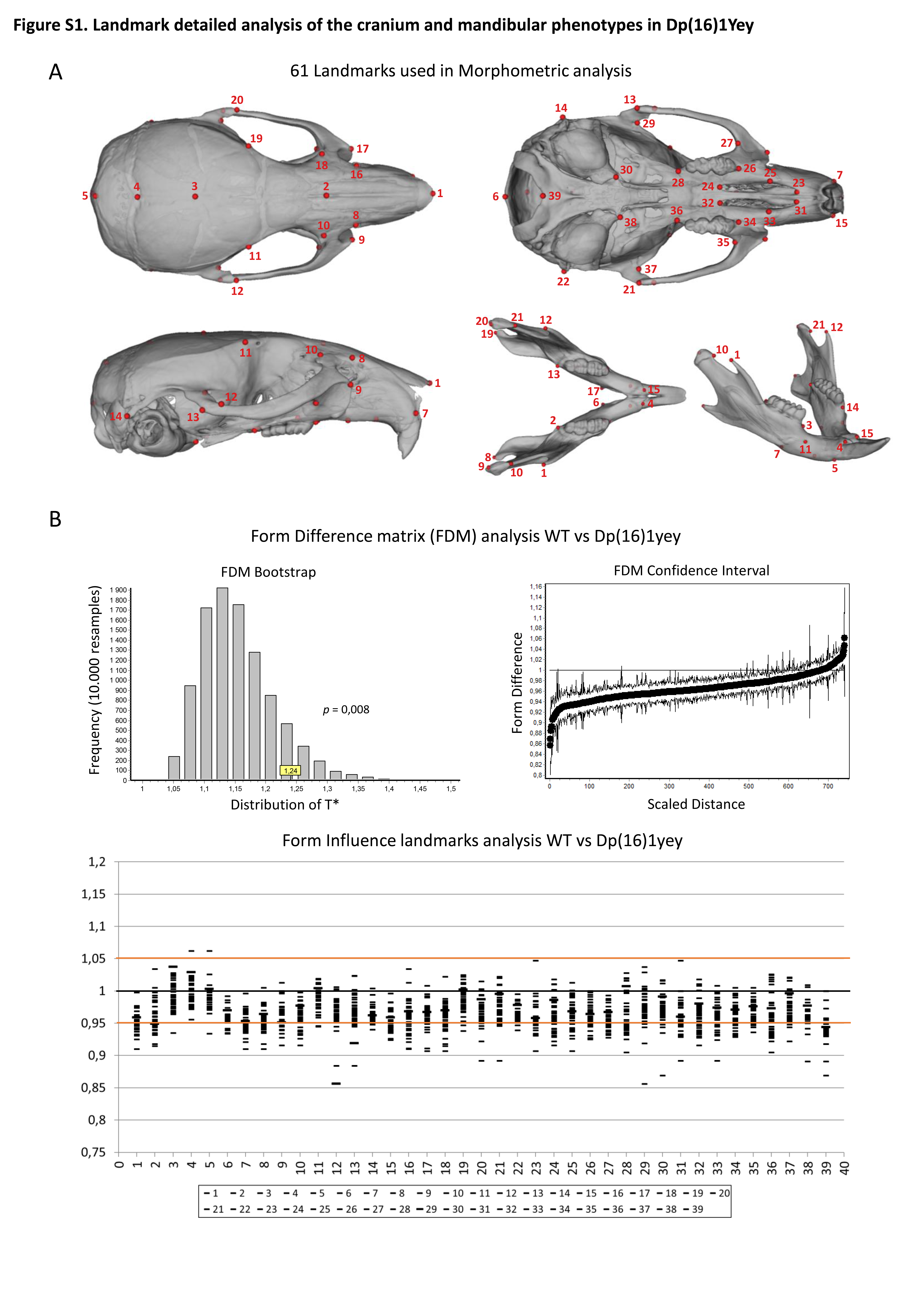

Supplement: S1 Fig — (A) 3D model of wild-type mouse sample done with 3DSlicer software, showing the 61 Landmarks used in the CF analysis of Dp(16)1Yey (and in all the other DS models). 39 in the cranium and 22 in the mandible. (B) Form difference matrix analysis: FDM Bootstrap with 10,000 iterations showing significant changes in Form (p = 0.008). The FDM confidence interval graph shows a decrease of more than 90% of the distances measured. Form Influence landmarks graphic, showing the landmarks that present a relative Euclidean distance > 1.05 or < 0.95 (outside of the confidence interval 97,8%, red lines) and a general reduction of all dimensions. (TIF) [file pgen.1011873.s003.tif]

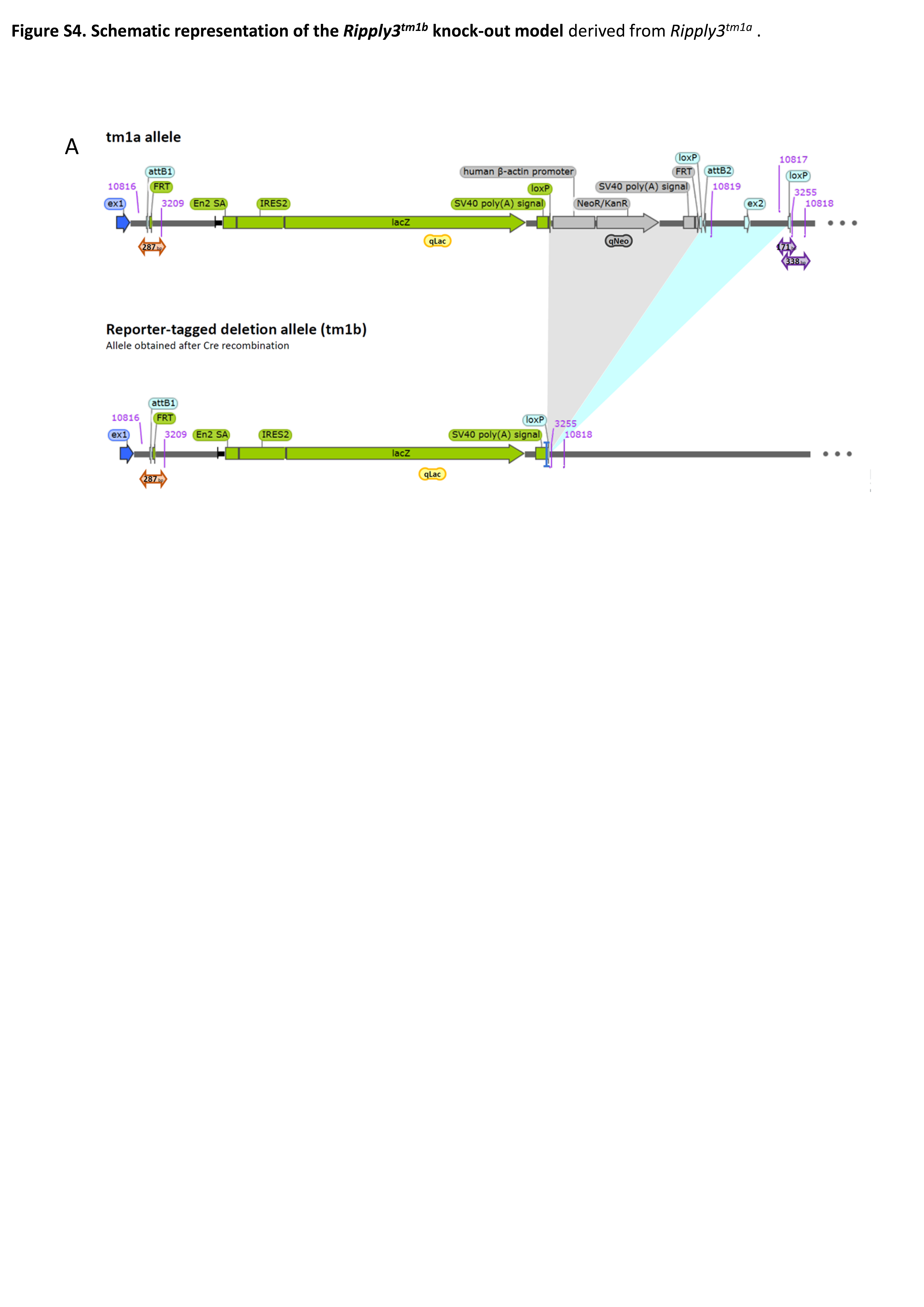

Supplement: S4 Fig — (TIF) [file pgen.1011873.s006.tif]
