# Supplementary material for: Ripply3 overdosage induces mid-face shortening through Tbx1 downregulation in Down syndrome models
Source: PLoS Genet. 2025 Sep 22;21(9):e1011873. doi: 10.1371/journal.pgen.1011873 (PMC12469710; doi:10.1371/journal.pgen.1011873)
Supplement: Attachment — Submitted filename: Rebuttal letter Ahumada et al Plos GenetV2_03-06-2025.docx [file pgen.1011873.s010.docx]

2^nd^ Rebutal


1) Please ensure that the CRediT author contributions listed for every co-author are completed accurately and in full.
At this stage, the following Authors/Authors require contributions: José Tomas Ahumada Saavedra, Claire Chevalier, Agnes Bloch Zupan, and Yann Herault. Please ensure that the full contributions of each author are acknowledged in the "Add/Edit/Remove Authors" section of our submission form.
The list of CRediT author contributions may be found here: <https://journals.plos.org/plosgenetics/s/authorship#loc-author-contributions>

Response: The Credit author contributions was inserted in the text and now was introduced in the "Add/Edit/Remove Authors" section of our submission form.

2) Please include a completed 'Competing Interests' statement in the text box when submitting your production task, including any COIs declared by your co-authors, written in full sentences. If you have no competing interests to declare, please state "The authors have declared that no competing interests exist". You may also provide an updated statement via email.

Response: we completed 'Competing Interests' statement in the text box

3) We note that you have uploaded Table 1 as other file. Tables should not be uploaded as individual files. Please remove these files and include the Tables in your manuscript file as editable, cell-based objects. For more information about how to format tables, see our guidelines:
<https://journals.plos.org/plosgenetics/s/tables>

Response: Corrected

4) We do not publish any copyright or trademark symbols that usually accompany proprietary names, eg ©, ®, or TM (e.g. next to drug or reagent names). Therefore please remove all instances of trademark/copyright symbols throughout the text, including: SuperScript™, VILO™, ZEN™, on page no 11, and Plus™ on page no 13.

Response: Removed

5) Thank you for stating "Mice were housed under specific pathogen-free (SPF) conditions and were treated in compliance with the animal welfare policies of the French Ministry of Agriculture (law 87 848).

Please include:
i) The full name(s) of the Institutional Review Board(s) or Ethics Committee(s)
ii) The approval number(s), or a statement that approval was granted by the named board(s).

Response: French regulations on the euthanasia of animals used in scientific experimentation result from the transposition of European Directive 2010/63/EU. Approved methods (gas inhalation, lethal injection, etc.) are defined by joint ministerial order of the ministers of agriculture, research and defense ([Décret n° 2013-118 du 1er février 2013 relatif à la protection des ...).](https://www.legifrance.gouv.fr/jorf/id/JORFTEXT000027037840)

In the current study, we have not done experiment on live animals and we euthanatized the mice **using standard validated and approved humane killing procedure** , as described in the text with the sentence after the one for the compliance statement. "Mice were housed under speciﬁc pathogen-free (SPF) conditions and were treated in compliance with the animal welfare policies of the French Ministry of Agriculture (law 87 848). **For each mouse line, about ten littermates by each genotype, DS, and wild-type (WT) were collected after euthanasia using standard validated and approved humane killing procedure (n = 180) in our facility approved for breeding and using animals for scientific purposes under number: D 67-218-40 (France)**.” Because we were using standard euthanasia, we did not have to apply for ethical approval according to the French regulations. Nevertheless, we took care not to overproduce samples for this study, keeping to the minimal number needed for relevant statistical analysis.

6) We have noticed that you have uploaded Supporting Information files, but you have not included a list of legends. Please add a full list of legends for your Supporting Information files after the references list.

Response: We have included the supplementary figures in the “supplementary information” file but we labelled it “supplementary figures. Thus, we made now the modification accordingly and remove the supplementary figures from the file as they are provided separately, and inserted a Legend to supplementary figures paragraph page 3

7) Some material included in your submission may be copyrighted. According to PLOSu2019s copyright policy, authors who use figures or other material (e.g., graphics, clipart, maps) from another author or copyright holder must demonstrate or obtain permission to publish this material under the Creative Commons Attribution 4.0 International (CC BY 4.0) License used by PLOS journals. Please closely review the details of PLOSu2019s copyright requirements here: PLOS Licenses and Copyright. If you need to request permissions from a copyright holder, you may use PLOS's Copyright Content Permission form.
Please respond directly to this email and provide any known details concerning your material's license terms and permissions required for reuse, even if you have not yet obtained copyright permissions or are unsure of your material's copyright compatibility. Once you have responded and addressed all other outstanding technical requirements, you may resubmit your manuscript within Editorial Manager.
Potential Copyright Issues:

Response: Can you be more precise here so that we can answer your question?

i) Figures 1, 4, and Figure S1. Please confirm whether you drew the images / clip-art within the figure panels by hand. If you did not draw the images, please provide (a) a link to the source of the images or icons and their license / terms of use; or (b) written permission from the copyright holder to publish the images or icons under our CC BY 4.0 license. Alternatively, you may replace the images with open source alternatives. See these open source resources you may use to replace images / clip-art:
- https://commons.wikimedia.org
- https://openclipart.org/.

Response: All the images that are represented in figures 1, 4 and S1 have been generated by us from the visualisation and analysis software.

8) Thank you for stating "The morphological data are deposited in Zenodo under the doi: 10.5281/zenodo.13639386"

Response: Thank you

Please note that, though access restrictions are acceptable now, your entire minimal dataset will need to be made freely accessible if your manuscript is accepted for publication. This policy applies to all data except where public deposition would breach compliance with the protocol approved by your research ethics board. If you are unable to adhere to our open data policy, please kindly revise your statement to explain your reasoning and we will seek the editor's input on an exemption.

9) Please amend your detailed Financial Disclosure statement. This is published with the article, therefore should be completed in full sentences and contain the exact wording you wish to be published.
i). State what role the funders took in the study. If the funders had no role in your study, please state: “The funders had no role in study design, data collection and analysis, decision to publish, or preparation of the manuscript.”

Response: the financial statement was introduced in the manuscript and is now also put in your submission portal.

“The funders had no role in study design, data collection and analysis, decision to publish, or preparation of the manuscript”

ii). If any authors received a salary from any of your funders, please state which authors and which funders.

Response: this is stated “JT A received funding from the National Agency for Research and Development (ANID)/Scholarship Program/DOCTORADO BECAS CHILE/2020- 72210028.”

10) Please ensure that the funders and grant numbers match between the Financial Disclosure field and the Funding Information tab in your submission form. Note that the funders must be provided in the same order in both places as well.
Currently, " EUR IMCBio (ANR-17- EURE-0023)" is missing from the Funding Information and also the order is different in both the places

Response: Done

**First REBUTALL LETTER with response to each point raised by the editor and reviewers**

1) Please ensure that the CRediT author contributions listed for every co-author are completed accurately and in full.
At this stage, the following Authors/Authors require contributions: José Tomas Ahumada Saavedra, Claire Chevalier, Agnes Bloch Zupan, and Yann Herault. Please ensure that the full contributions of each author are acknowledged in the "Add/Edit/Remove Authors" section of our submission form.
The list of CRediT author contributions may be found here: <https://journals.plos.org/plosgenetics/s/authorship#loc-author-contributions>

Response:

2) We ask that a manuscript source file is provided at Revision. Please upload your manuscript file as a .doc, .docx, .rtf or .tex. If you are providing a .tex file, please upload it under the item type u2018LaTeX Source Fileu2019 and leave your .pdf version as the item type u2018Manuscriptu2019.

Response: Done

3) Please provide an Author Summary. This should appear in your manuscript between the Abstract (if applicable) and the Introduction, and should be 150-200 words long. The aim should be to make your findings accessible to a wide audience that includes both scientists and non-scientists. Sample summaries can be found on our website under Submission Guidelines:
<https://journals.plos.org/plosgenetics/s/submission-guidelines#loc-parts-of-a-submission>

Response here is the Author Summary of 168 words that has been inserted between the Abstract and the Introduction :

An extra copy of chromosome 21 causes down syndrome (DS) and leads to intellectual disability and distinct facial features. To understand which genes drive these traits, we have engineered mouse models that mimic the DS genetic changes. By studying these mice, we identified regions of mouse chromosome 16, equivalent to human chromosome 21, that are associated with the head and face differences in Down syndrome (DS). We pinpointed two genes, *Dyrk1a* and *Ripply3*, whose increased activity disrupts normal facial development by altering the growth of specific cells during embryonic development. Notably, reducing Ripply3 activity in these mice corrected some facial abnormalities, highlighting its crucial role. These findings clarify how extra genetic material leads to DS features and suggest new therapeutic targets. The study also highlights the importance of animal models in elucidating the genetic and developmental basis of human conditions, such as DS. Additionally, the mechanism involving *Ripply3* connects DS with DiGeorge syndrome, another disorder with similar facial changes, suggesting shared pathways behind some features of both conditions.

) We do not publish any copyright or trademark symbols that usually accompany proprietary names, eg ©,  ®, or TM  (e.g. next to drug or reagent names). Therefore, please remove all instances of trademark/copyright symbols throughout the text, including:

Response: Correction were done for ®, on pages: 7, 9, 18, and 32 and for TM on pages 10 and 12.

5) Thank you for including an Ethics Statement for your study. Please include:
i) The full name(s) of the Institutional Review Board(s) or Ethics Committee(s).

Response: You must consider that we do not need to have an ethical approval for the studies described here which was done on skeleton samples obtained after using recognized procedure for euthanasia. This is acknowledged in the paragraph “Ethics Statement and Adult cohorts generated” page 7, where you can find “after euthanasia using standard validated humane killing procedure (n = 180) in our facility approved for breeding and using animals for scientific purposes under number: D 67-218-40 (France). “

“Ethics Statement and Adult cohorts generated

Mice were housed under speciﬁc pathogen-free (SPF) conditions and were treated in compliance with the animal welfare policies of the French Ministry of Agriculture (law 87 848). For each mouse line, about ten littermates by each genotype, DS, and wild-type (WT) were collected after euthanasia using standard validated humane killing procedure (n = 180) in our facility approved for breeding and using animals for scientific purposes under number: D 67-218-40 (France). We tried to have balanced males and females in the cohorts. As a major genotype effect compared to sex was previously described elsewhere independently (Redhead et al. 2023), and optimize the number of animals used in this project, to follow the 3Rs rule, we decided to check whether the sex effect was insignificant compared to genotype in the Dp(16)1Yey line. Thus, For the Dp(16)1Yey line, six females plus five males for the dup carrier and six males plus three females for control were used. As we did not find sexe as a significant variable, we then continued mainly without considering sexe, with sometimes more female individuals collected than males, because males were used to breed the lines. “

6) Please upload all main figures as separate Figure files in .tif or .eps format. For more information about how to convert and format your figure files please see our guidelines: 
<https://journals.plos.org/plosgenetics/s/figures>

Response: OK

7) We notice that your supplementary Figures, and Tables are included in the manuscript file. Please remove them and upload them with the file type 'Supporting Information'. Please ensure that each Supporting Information file has a legend listed in the manuscript after the references list.

Response: done and the table is now provided separately as a supporting information with the legend at the end of the text page 34:

**“LEGEND TO TABLES**

**Table 1.** **Summary of the CF phenotypes observed in DS models**. Table expressing the level of changes found in skulls and mandibles: Mild, Strong and No significative (NS). “"

8) Some material included in your submission may be copyrighted. According to PLOSu2019s copyright policy, authors who use figures or other material (e.g., graphics, clipart, maps) from another author or copyright holder must demonstrate or obtain permission to publish this material under the Creative Commons Attribution 4.0 International (CC BY 4.0) License used by PLOS journals. Please closely review the details of PLOSu2019s copyright requirements here: PLOS Licenses and Copyright. If you need to request permissions from a copyright holder, you may use PLOS's Copyright Content Permission form.
Please respond directly to this email and provide any known details concerning your material's license terms and permissions required for reuse, even if you have not yet obtained copyright permissions or are unsure of your material's copyright compatibility. Once you have responded and addressed all other outstanding technical requirements, you may resubmit your manuscript within Editorial Manager. 
Potential Copyright Issues:
i) Figures 3C, 4C, and S1. Please confirm whether you drew the images / clip-art within the figure panels by hand. If you did not draw the images, please provide (a) a link to the source of the images or icons and their license / terms of use; or (b) written permission from the copyright holder to publish the images or icons under our CC BY 4.0 license. Alternatively, you may replace the images with open source alternatives. See these open source resources you may use to replace images / clip-art:
- <https://commons.wikimedia.org>
- <https://openclipart.org/>.

Response: The Embryo image in figure 3C was from BioRender. As we do not have copyright, and we did not find a relevant image in other public library, we removed it.

9) We note that your Data Availability Statement is currently as follows: "All the data are available as indicated in the manuscript.". Please confirm at this time whether or not your submission contains all raw data required to replicate the results of your study. Authors must share the “minimal data set” for their submission. PLOS defines the minimal data set to consist of the data required to replicate all study findings reported in the article, as well as related metadata and methods (<https://journals.plos.org/plosone/s/data-availability#loc-minimal-data-set-definition>).
For example, authors should submit the following data: 
1) The values behind the means, standard deviations and other measures reported;
2) The values used to build graphs;
3) The points extracted from images for analysis..
Authors do not need to submit their entire data set if only a portion of the data was used in the reported study.
If your submission does not contain these data, please either upload them as Supporting Information files or deposit them to a stable, public repository and provide us with the relevant URLs, DOIs, or accession numbers. For a list of recommended repositories, please see <https://journals.plos.org/plosone/s/recommended-repositories>. 
If there are ethical or legal restrictions on sharing a de-identified data set, please explain them in detail (e.g., data contain potentially sensitive information, data are owned by a third-party organization, etc.) and who has imposed them (e.g., an ethics committee). Please also provide contact information for a data access committee, ethics committee, or other institutional body to which data requests may be sent. If data are owned by a third party, please indicate how others may request data access.

Response: all the data will be made publicly available through the Zenodo resource center [https://zenodo.org/records/13639386 with the DOI 10.5281/zenodo.13639386](https://zenodo.org/records/13639386%20with%20the%20DOI%2010.5281/zenodo.13639386) as soon as the publication is accepted and we will link to the paper.

The reference to the dataset is indicated in the paragraph page 25.

**Data availability**

The morphological data are deposited in Zenodo under the doi: 10.5281/zenodo.13639386

10) Please amend your detailed Financial Disclosure statement. This is published with the article. It must therefore be completed in full sentences and contain the exact wording you wish to be published.
1) State what role the funders took in the study. If the funders had no role in your study, please state: "The funders had no role in study design, data collection and analysis, decision to publish, or preparation of the manuscript."

The mention "The funders had no role in study design, data collection and analysis, decision to publish, or preparation of the manuscript." has been inserted in the web portal

See the paragraph page 25.

**Funding**

This work of the Interdisciplinary Thematic Institute IMCBio, as part of the ITI 2021-2028 program of the University of Strasbourg, CNRS and Inserm, was supported by IdEx Unistra (ANR-10-IDEX-0002), SFRI-STRAT’US project (ANR 20-SFRI-0012), INBS PHENOMIN (ANR-10-INBS-07) and EUR IMCBio (ANR-17-EURE-0023) under the framework of the French Investments for the Future Program. YH received funding from funding from the European Union’s Horizon 2020 research and innovation program under grant agreement No 848077 for GO-DS21. JT A received funding from the National Agency for Research and Development (ANID)/Scholarship Program/DOCTORADO BECAS CHILE/2020- 72210028. The funders had no role in study design, data collection and analysis, decision to publish, or preparation of the manuscript

11) Please ensure that the funders and grant numbers match between the Financial Disclosure field and the Funding Information tab in your submission form. Note that the funders must be provided in the same order in both places as well.

Response: Confirmed

12) Please provide a completed 'Competing Interests' statement, including any COIs declared by your co-authors. If you have no competing interests to declare, please state "The authors have declared that no competing interests exist". Otherwise please declare all competing interests beginning with the statement "I have read the journal's policy and the authors of this manuscript have the following competing interests:"

The sentence "The authors have declared that no competing interests exist" has been inserted in the paragraph page 25.

**Conflict of interest statement**

The authors declare no competing interests.

**Reviewers' comments:**

Reviewer's Responses to Questions

Reviewer #1: This study by Herault and colleagues capitalizes on mouse models of Down syndrome to identify driver genes for the craniofacial phenotype. Their approach to using chromosomal segments in the trisomy portion of human chromosome 21 is an elegant extension of the previous approach applied to human chromosome 22q11 (PMID: 16365290; PMID: 23917946; PMID: 19617637;PMID: 10545603; PMID: 16684884). The authors are also commended for rigorously controlling the genetic backgrounds of mouse models they used and generated, as this issue has compromised many mouse studies (PMID: 29369447).
Their analysis confirmed Dyrk1a and, additionally, discovered Ripply3 as driver genes for the phenotype. Remarkably, the authors also identified Tbx1 as a potential intermediate molecule, down regulation of which also has been shown to reduce the craniofacial phenotype. This is another clear evidence that even large CNVs contain single driver genes, refuting a claim made by some, based on a small-scale analysis, that each large CNV does not contain single driver genes.
**As is often the case with work coming from this lab, the study is well designed, data are adequately analysed and interesting, and the results are important and have broad implications. This reviewer has only minor suggestions for improving this excellent work.**

1. The data presented in Figure 3BC show considerably variable variance among gene groups. It should be described in the figure legend if the homogeneity of variance was or was not violated among groups, if the former, what non-parametric tests were used for statistical analysis.

Responses to 1: We assessed the normality of the RT-ddPCR data distributions using the Shapiro-Wilk test (α = 0.05), and all ddPCR expression datasets satisfied the normality assumption. Consequently, we performed statistical comparisons using a two-tailed unpaired t-test. This information has now been included in the legend of Figure 3.

**Figure 3. Genetic mapping identifies a new chromosomic region and dosage-sensitive genes for the DS craniofacial phenotype. (A)** Scheme of the relative position of the new mouse and rat models in HSA21, and the new chromosomic region of interest for DS CF phenotype (red square). **(B)** RT-ddPCR results graphics for (left) Mouse model Dp(16)1Yey E11.5 in the left, and (right) rat DS Dp(RNO11) E12.5. RNA was isolated from the branchial arch region of trisomic and euploid controls (n = 10 per group). Relative gene expression ratios (trisomic/euploid) are shown for *Ripply3*, *Tbx1* and *Dyrk1a*.After confirming normality via Shapiro-Wilk test (α = 0.05), statistical comparisons were performed using a two-tailed unpaired t-test. Significance thresholds: ****, p<0.0001; ***, p<0.001; ** for p<0.01; *, p<0.05. Significant overexpression of triplicated genes *Dyrk1a* and *Ripply3*, alongside downregulation of *Tbx1*, was observed in both species (vs. euploid controls). **(C)** Edu, PH3, and Hoechst unrevealed defects in the proliferation and mitosis of the NCC derivates in the 1st branchial arch during craniofacial development in Dp(16)1Yey at E.5. Here too, normality was confirmed by Shapiro-Wilk test (α = 0.05) and statistical comparisons were performed using a two-tailed unpaired t-test. In the right graphic, the proliferation (***, p<0.000) and mitotic (***, p<0.000) indexes were significantly reduced in Dp(16)1Yey.

2. The images in Figure 3C are not of good quality, and EdU-positive nuclei are not visible. Higher-quality images should be used.

Response to 2: We provided new images of better quality for the figure 3C that are taken from cryosection of E9.5 mouse embryo branchial arches observed using fluorescence imaging for EdU, PH3 and Hoetsch.

3. Data in Figure 3 are consistent with their hypothesis, but it does not directly demonstrate the causative role of Tbx1 as an intermediate variable between Dyrk1a/Ripply3 and craniofacial phenotypes. This limitation should be more fully discussed.

Response to 3. We agree that Data in Figure 3 are consistent with our hypothesis, but it does not directly demonstrate the causative role of *Tbx1* as an intermediate variable between Dyrk1a/Ripply3 and craniofacial phenotypes. We have indicated this in the discussion where we presented evidence from other groups that RIPPLY3 downregulates Tbx1 expression and point to the role of *Tbx1* in DiGeorge Syndrome CF features.

4. Tbx1 gene dose alterations contribute to the structural phenotypes of the brain (PMID: 34737458; PMID: 39463450) and affects the function of adult stem cells (PMID: 23917946). The authors might want to discuss this point.

Response to 4: We thank the reviewer for his/her recommendation and we highlighted these points in the last paragraph of the discussion on page 24

“Interestingly, *Tbx1* down-regulation in DGS is known to affect the structure of the brain with an overall decrease in myelin in the fimbria, probably through reducing oligodendrocyte generation (Hiramoto et al. 2022), in the amygdaloid complex and close cortical regions (Hiramoto et al. 2025). Concomitantly, *Tbx1* haploinsufficiency causes phenotypes in social interaction and communication, a tendency toward repetitive behaviour, impaired working memory, slower acquisition of spatial memory, and reduced cognitive flexibility (Hiramoto et al. 2022; 2011; Hiroi et al. 2013). »

Reviewer #2: This manuscript addresses the genetic causes of the craniofacial defects in Down syndrome (DS). The syndrome results from trisomy of chromosome 21 (Hsa21) and is thus most likely due to increased dosage of one or more of the genes on Hsa21. The authors use a mouse model of DS, Dp(16)1Yey, which has an extra copy of a region of mouse chromosome 16 (Mmu16) that is orthologous to Hsa21. They confirm earlier studies, showing that adult Dp(16)1Yey mice have altered craniofacial alterations including a smaller cranium and mandible, brachycephaly and midfacial hypoplasia. They then extend the analysis to a new series of 7 mouse strains with duplications of different parts of the Mmu16 region, in order to map the location of genes whose increased dosage causes the defects. They conclude that there are three areas of Mmu16 that contribute to the phenotype. In addition, they show that two of the causative genes are Dyrk1a and Ripply3. Of these, previous studies had already implicated increased dosage of Dyrk1a in causing craniofacial changes in DS. However, the identification of Ripply3 is a novel finding. Overall, this is an interesting study using suitable approaches to find the genes that cause DS phenotypes. However, I have several concerns, including that the manuscript is hard to follow in many places.

Major points.

1. The authors report that they find significant changes in Dp(16)1Yey mice in both form and shape. What do the authors consider to be the difference between form and shape?

Response: We have provided only a summary of the morphometric analysis in the Materials and Methods section because those methods are standards and published (Lele and Richtsmeier 2001). To clarify, "form" refers to the combination of size and shape of a geometric object in any orientation and location, while "shape" describes the geometry of the object once it has been standardized for size, position, and orientation.

2. How do the authors determine that there are statistically significant changes between Dp(16)1Yey mice and their WT controls?

Response: As stated in the Materials and Methods and presented in the publication by Lele and Richtsmeier (2001) the Morphological differences between groups can be pinpointed to specific linear distances on an object through pairwise comparisons of mean form or shape matrices, followed by bootstrapping to estimate the significance of these differences (Lele and Richtsmeier 2001). In this study, two tests were done for each group of samples, first to analyze the form of the skull and mandibles with form difference matrix (FDM) and then the shape with the shape difference matrix (SDM).

3. Do the authors assess size changes separately from shape changes? I understand that they are measuring distances between landmarks shown in S1A. However, differences could be due to changes in size or shape or both. It would be important to distinguish these two distinct effects. Can size differences be regressed out to determine if there are shape differences as well?

Response: you are right when analysing the "form" we have the combination of size and shape, but when we are analysing the "shape" describes the geometry of the object is now standardized for size, position, and orientation. Thus, we can discriminate shape changes form size. This is what we have been doing in this analysis with the classical Euclidean Distance Matrix Analysis (EDMA).

4. Figure S1. What is the meaning of the graphs in B? They are not explained sufficiently well for the reader to understand. What is being plotted in each of these? What is the meaning of the x- and y-axes of the histogram? What is Form difference and Form Confidence Interval? Some of the labels are very small and hard to read. Same applies to Figures S2, 4A and B, which are also not understandable.

Response: The graphs in B are classical graphs calculated during the EDMA analysis representing on the Upper part the form difference matrix (FDMà analysis of the WT versus the Dp(16)1Yeh. The upper graph on the left “FDM represents the Bootstrap method for obtaining confidence intervals while the upper graph on the right shows the distance interval calculated between the 39 landmarks in the skull (about 741 Euclidian distance between two distinct landmark) ranked by their ratio Distance(I,J)WT/Distance(I,J)Dp, no change will be 1.

For the bootstrap analysis: Suppose that the estimated form difference matrix determines that a particular distance is larger in object B as compared to object A. To know there is statistically significant difference, basic statistics tells us that if we have a very large number of observations, or if the biological variability is very small, we can put greater trust in the estimated form difference matrix than if we had a small sample size and/or large variability. For this we used Bootstrapping, this is a re-sampling technique used to estimate the sampling distribution of a statistic by repeatedly sampling (with replacement) from the original dataset.

Once obtained the landmark coordinate, Form Matrices (FM) are calculated for each individual (WT:A vs DS:B). Then the two matrices are analyse using form difference matrix (FDM) with the following equation: FDM(B,A)=[FM(B)/FM(A)], computing the mean forms and comparing them using the *T* statistic. The X-axe represents the frequency of the re-samples and the Y-axe represent the distribution of T. Finally, a bootstrap version of the test statistic T, called T* is computed, with this data is possible to calculate the statistical difference between the two samples (p value).

The FDM confidence interval: Each FDM(B,A) calculated from a Bootstrap sample is written as a vector with K(K-1) /2 entries. The collection of all FDM(B,A) obtained in this way is written as a matrix that has K(K-1) /2 rows and X columns. Each FDM is sorted according to the landmarks that define the linear distance. Each column is a form difference matrix in vector format obtained with FDM(B,A)=[FM(B)/FM(A), and each row represents X form difference ratios for a linear distance between a specified pair of landmarks.

To obtain a confidence interval for each linear distance, the ratios in each row are sorted in increasing order. The 100(1-α)% confidence interval is delimited by removing the first α/2 % and the last α/2 % of the sorted entries. The minimum and maximum entries remaining in that row constitute the lower and upper confidence limits for that particular linear distance. If the interval of entries spanning the lower and upper confidence limits contains the value 1, then it is likely that the particular distance is not different in the two populations. The interval also provides some idea as to the range of values that particular ratios might take. Similar statements may be made separately for each row using the values obtained to estimate the confidence interval for each linear distance.

The morphological data are deposited in Zenodo under the doi: 10.5281/zenodo.13639386

5. What data input is used for the PCA analysis shown in Figure 1A? Is it distances between landmarks? Or between landmarks and the centroid? Or something else? Please explain clearly. Are the differences between WT and Dp(16)1Yey significant? What statistical test is used for this? Provide suitable metrics, e.g. p-values.

Response : PCA analysis was done in the MorphoJ software. The data used corresponds to the 3D coordinates of each sample (format: the data lines contain a label identifying the specimen as the first entry, and then the landmark coordinates in the order x, y, z, x, y, z,..., x, y, z for three-dimensional data. Text files can be found in the supplementary data). Then Procrustes superimposition is performed (Procrustes fit) to generate a covariance matrix (a square matrix giving the covariance between each pair of elements of a given random vector), to finally perform the principal component analysis. The statistical differences were made in EDMA, with the FDM and SDM tests. Graphics with the statistical significance can be found in the S1.B figure.

6. The analysis of the multiple mouse strains in is hard to follow. The authors analyze 7 new mouse strains and present the data in Figures 2, S2, S3. A genomic map showing all the strains would be very useful at this point. In Figure 3A they authors show a map but it only contains 4 of these strains. A map with all the strains does not appear until Figure 5A, three figures later. And even this map is hard to read - the strains are not listed in either genomic order or in numerical order, with labels sometimes on the left and sometimes on the right, making it hard to find any specific strain.

Response : We understand and acknowledge that the analysis of the multiple strain is complex. Now we introduced the 10 mouse models in figure 3A . We preserved the figure 2A with the exact position of the duplicated segments for the new models and the analysis done model by model for the cranium with complementary data from the EDMA in figure S2 and the mandible in figure S3. We tried to put the label of the strain on the same side but we could not for one in fig 3A and 2 in fig 5A.

7. On page 16 the authors announce that Ripply3 is a promising target for a gene that may be causing the craniofacial changes. However, they explain nothing about their logic for picking this gene - this is the first mention of the gene in the manuscript. Why was it chosen? Why were other genes ignored?

Response : We agree that we did not detailed how we selected Ripply 3 for CF changes in DS models because we considered some bibliographic references. With the results of the morphometric analysis performed in this study, on the new rats models and new panel of down syndrome mouse models (plus the existing Dp(16)1yey, Tg(Dyrk1a)YaH). We identified and proposed a new chromosomic region in the Mmu16 as responsible for the craniofacial phenotype found in DS. Inside this new region there are 15 genes: *Setd4, Cbr1, Gm5678, Cbr3, Dop1b, Morc3, Chaf1b, Cldn14, Sim2, Hlcs, Ripply3****,*** *Pigp, Ttc3, Vps26c and Dyrk1a****.*** To isolate candidate genes for the craniofacial development we analyze a dataset from the FaceBase Consortium ‘Temporal analysis of ectoderm and mesenchyme gene expression in the developing mouse facial prominences’ (Hong Li 2017), where RNA-seq was performed in the following wild-type tissues: [Ectoderm of mandibular part of first pharyngeal arch](https://www.facebase.org/chaise/record/#1/vocab:anatomy/RID=1-4FFR?pcid=record&ppid=2bm721s52otf1o8t1hyj2a0l), [Ectoderm of maxillary process](https://www.facebase.org/chaise/record/#1/vocab:anatomy/RID=1-4FFT?pcid=record&ppid=2bm721s52otf1o8t1hyj2a0l), [head mesenchyme](https://www.facebase.org/chaise/record/#1/vocab:anatomy/RID=1-XP12?pcid=record&ppid=2bm721s52otf1o8t1hyj2a0l), [mesenchyme of frontonasal process](https://www.facebase.org/chaise/record/#1/vocab:anatomy/RID=1-4FCP?pcid=record&ppid=2bm721s52otf1o8t1hyj2a0l), [Mesenchyme of mandibular part of first pharyngeal arch](https://www.facebase.org/chaise/record/#1/vocab:anatomy/RID=1-4FG6?pcid=record&ppid=2bm721s52otf1o8t1hyj2a0l), [Mesenchyme of maxillary process](https://www.facebase.org/chaise/record/#1/vocab:anatomy/RID=1-4FG8?pcid=record&ppid=2bm721s52otf1o8t1hyj2a0l), [Nasal placode](https://www.facebase.org/chaise/record/#1/vocab:anatomy/RID=1-4FGA?pcid=record&ppid=2bm721s52otf1o8t1hyj2a0l), [olfactory epithelium](https://www.facebase.org/chaise/record/#1/vocab:anatomy/RID=1-XSQ6?pcid=record&ppid=2bm721s52otf1o8t1hyj2a0l), [olfactory pit](https://www.facebase.org/chaise/record/#1/vocab:anatomy/RID=1-XERM?pcid=record&ppid=2bm721s52otf1o8t1hyj2a0l) and [oral ectoderm](https://www.facebase.org/chaise/record/#1/vocab:anatomy/RID=1-GM52?pcid=record&ppid=2bm721s52otf1o8t1hyj2a0l) at 3 different stages, E10.5, E11.5 and E12.5. Thanks to this dataset we identified a list of 12 genes in the Mmu16 with a significant expression in the developing craniofacial tissues: *Cbr1, Cbr3, Chaf1b, Dyrk1a****,*** *Ets2*, *Hlcs, Kcnj15, Morc3, Gm56, Ripply3****,*** *Sim2****,****Gm5678.* We overlapped these genes with the genes of our new proposed chromosomic region, creating a new list of with the following genes: *Cbr1, Gm5678, Cbr3,**Morc3, Chaf1b, Sim2, Hlcs, Ripply3 and Dyrk1a.* To reduce this list, bibliographic research was done in MGI, IMPC and OMIM, where only *Sim2*, *Ripply3* and *Dyrk1a* showed roles and phenotypes in mouse models in the craniofacial structure. Dirst, we selected *Dyrk1a* (Dual Specificity Tyrosine Phosphorylation Regulated Kinase 1A) with a strong bibliography supporting the role in the craniofacial tissues in DS mouse models in our team previous publications (Duchon and Herault, 2016; Atas-Ozcan et al. 2021; Duchon et al. 2022). Then we included *Ripply3* (Ripply Transcriptional Repressor 3), since its acts as a negative regulator of *Tbx1*, and is an already know gene involved in a strong craniofacial phenotype in DiGeorge/Velocardiofacial syndromes. Thus, we modified the introductory paragraph of the relevant section now in page 17.

**Candidate genes for the CF DS phenotype in DS rodent models**.

We focused on the new chromosomal region selected from the previous morphometric analysis, encompassing 15 genes between Setd4 –Dyrk1a genes (Fig 3A). Inside this region, based on scientific literature and a data set from the FaceBase Consortium (Hong Li 2017), we isolated a set of candidate genes. Among these genes, *Dyrk1a* (Dual Specificity Tyrosine Phosphorylation Regulated Kinase 1A) and *Ripply3* (*Ripply Transcriptional Repressor 3*) seemed promising targets for CF in DS. *Dyrk1a* overdosage in DS CF phenotype was already pointed out in several studies (Guedj et al. 2012; Redhead et al. 2023; McElyea et al. 2016; Starbuck et al. 2021) while *Ripply3* is a new interesting candidate. Indeed, several studies demonstrated Ripply3's role as a transcriptional repressor of *Tbx1* across species and developmental contexts (Janesick et al. 2012; Pane et al. 2018; Okubo et al. 2011). In addition, as presented in the introduction, *Tbx1* is the primary genetic driver of CF phenotypes in DiGeorge Syndrome. Thus, we hypothesize that *Ripply3* overdosage in DS contributes to CF phenotypes in mouse models through down-regulation of *Tbx1*.

8. On page 18 the authors cross Dp(16)1Yey mice to a Ripply3 KO allele to test if reducing the copy number of Ripply3 reverses the defects. They state that Dp(16)1Yey/Ripply3tm1b mice show significant shape changes in the skulls and mandibles compared to Dp(16)1Yey mice. Where is the data showing that the shape differences are significant?

Response: (now page 19) We compared the shapes using EDMA of Dp(16)1Yey and Dp(16)1Yey/Ripply3tm1b mouse models. The null hypothesis posits that the mean form of one population is a scaled version of the other (i.e., the ratios of all linear distances are equal to a constant). The inability to reject this null hypothesis indicates that the two samples of forms are similar in shape, differing only in size. The alternative hypothesis suggests that the two populations differ in form, meaning they are not merely scaled versions of one another. While differences in size exist, they vary across the linear distances considered. If the histogram indicates a bimodal distribution with a significant dip in the number of observations near zero, as shown in figure 4A, the null hypothesis should be rejected. The shape is statistically significantly different in both genotypes.

To ascertain the exact landmarks that produce this shape variation, we perform the influential landmarks analysis (Figure 4B), which allows for the identification of landmarks displaying the highest relative Euclidean distance difference (marked by the red circles). Two additional methods for visualising these changes are the warping technique illustrated in figure 4D and the shape difference analysis conducted with Vultus, represented in figure 4E. When comparing shapes using EDMA, the null hypothesis is that the mean form of one population is a scaled version of the other population (i.e., the ratios of all linear distances are equal to a constant). The inability to reject this null hypothesis would mean that the two samples of forms are similar in shape and differ only in size. The alternative hypothesis is that the two populations differ in form. In this case, the two populations are not merely scaled versions of one another. Differences in size exist but vary among the linear distances considered. If the histogram indicates a bimodal distribution with a large dip in the number of observations near zero, as in figure 4A, the null hypothesis should be rejected (The shape is statistically significant). To know the exact landmarks that generate this shape variation we perform the influential landmarks analysis (Figure 4B) where is possible to identify the landmarks with a higher relative Euclidean distance difference (in the red circles). Two other ways to visualize these changes are the warping technique in the figure 4D and in the shape difference analysis done with Vultus, figure 4E.

9. The PCA plots in Figure 4D show that the two genotypes overlap closely in shape. The legend says they are significantly different, but that seems surprising given the substantial overlap. What statistical test was used to show differences? Please include appropriate metrics, e.g. p-values.

Response: in figure 4D, the warping is the most informative part. They show that the Skull shape of the Dp(16)1Yey/ *Ripply3^tm1b^* when compared to the Dp(16)1Yey (last right image). We agree the PCA is not very informative. Indeed, it is based on the analysis of all the Euclidian distances calculated from the 39 landmarks used for the Skull, whereas only the 14 landmarks highlighted in 4C are involved in the rescue.

10. Top of page 19 the authors state that the heat map shows increased dimensions in all the bones in the red region. Which genotype is increased relative to which other? Is it Dp(16)1Yey or Dp(16)1Yey/Ripply3tm1b that is increased? This confusion is present in several places in the manuscript which should be edited carefully.

Response: Sentences was corrected in the end of page 20. “In the heat map, in red, we found the structures with increased dimensions in Dp(16)1Yey/Ripply3^tm1b^, that correspond to the bones located in the midface. In light blue, the structures that did not present significant changes (Fig. 4E).”

It should be noted that the analysis uses the average WT model as a reference, therefore the changes that we observe are in the average DS model analyzed. Observing in red the structures with a higher dimension in the DS model, and in blue the structures with a lower dimension (this applies to all the analyses made in this study).

Explanation added in Materials and methods, page 9, paragraph 3: “This analysis used the average WT model as a reference; therefore, the changes were observed in the average DS model analyzed. Indicated in red are the structures with a higher dimension in the DS model of interest, and in blue the structures with a lower dimension. “


Minor points.

11. Introduction. The authors state that Hsa21 has 671 genes, but then state that Ts65Dn mice are trisomic for 104 genes. I suspect that 671 refers to both coding and non-coding genes, whereas 104 refers to just coding genes. The authors should clarify this, and for consistency they might consider reporting numbers of the same type of genes for both human and mouse, e.g. just coding gene.

Response: thank for pointing, we agreed and we corrected the introduction to only indicate 222 protein coding genes according to the Ensembl database <https://www.ensembl.org/Homo_sapiens/Location/Chromosome?r=21:25570024-25670024>

12. In the abstract and the discussion, the authors state that brachycephaly means a wider skull. Brachycephaly usually refers to the skull being shorter front to back.

Response: We acknowledged that we should be more precise as brachycephaly is a term to describe a condition in which the head is abnormally short and broad, giving it a flattened appearance from front to back. We corrected the abstract and discussion accordingly.

Abstract (page 1):

The DS-specific CF features are an overall reduction in head dimensions (microcephaly), relatively wide and broad neurocranium (brachycephaly), …

Introduction (page 2)

DS-related CF dysmorphism includes an overall reduction in head dimensions (microcephaly), relatively short and broad neurocranium (brachycephaly),…

Discussion (page 21)

The DS CF phenotype typically encompasses microcephaly, a small midface, a reduced mediolateral orbital region, reduced bizygomatic breadth, a small maxilla, brachycephaly (a relatively wide and broad neurocranium), and a small mandible (Olson et al. 2004).

13. Methods. Mouse strains are reported to have all been maintained on C57BL/6J. Was the Ts66Yah mouse also maintained on C57BL/6J? If not, could that be a reason why Ts66Yah skulls lie in a different part of the PCA plots from Dp(16)1Yey and others?

Response: The reviewer is right: the Ts66Yah mouse is not maintained on C57BL/6J. We can only conclude from the comparative analysis in fig 5 that the hybrid genetic background has a lower influence than the genetics of the models. Indeed, all the controls (noted WT) from B6 and B6C3B are grouped together . It is important to note and we had this information to the page 20 3^rd^ paragraph:

“As shown in Fig. 5, all the wild-type littermate controls, from the B6C3B F1 hybrid genetic background (for Ts66Yah) and the B6J pure background (for all the other models), clustered together for the cranium and the mandible, with a higher variation, probably due to the altered position of some landmarks. “

14. Results, page 13, paragraph 3. The final 2 sentences are repeated on page 14 paragraph 2.

Response: Correction made. Repeated sentence “For a more detailed investigation of the patterns of landmark displacements and their dimensionality, we employed Principal Component Analysis (PCA). In skull and mandible, the PCA of Dp(16)1Yey showed significant differences in the dimensionality versus the WT group (Figure 1A).” Eliminated on page 14, paragraph 3.

15. Figures S4 and S5 are not cited in the manuscript.

Response: Thank you for noticing these errors. Now both figures are page 19 for S4 and 20 for S5

16. Page 20, line 9. The authors state that Dp(16)1Yey mice have a complete duplication of Mmu16. This is not correct. They have a duplication of only 22.9 Mb of Mmu16.

Response: we corrected the sentence now page 21: “We used Dp(16)1Yey to study this characteristic phenotype. This model carries a complete duplication of the Mmu16 region homologous to Hsa21 (Li et al. 2007) »
