# Supplementary material for: Ripply3 overdosage induces mid-face shortening through Tbx1 downregulation in Down syndrome models
Source: PLoS Genet. 2025 Sep 22;21(9):e1011873. doi: 10.1371/journal.pgen.1011873 (PMC12469710; doi:10.1371/journal.pgen.1011873)
Supplement: Attachment — Submitted filename: Rebuttal letter 2.docx [file pgen.1011873.s011.docx]

PGENETICS-D-25-00125R1
Ripply3 overdosage induces mid-face shortening through Tbx1 downregulation in Down syndrome models.
PLOS Genetics

Subject: Re: Manuscript Revision Invitation – PLOS Genetics

Dear Dr. Bosco,

Dear Dr. Dudley,

Thank you for your message and for the thoughtful consideration of our manuscript. We appreciate the constructive feedback provided by you and the reviewers, and we are pleased to have the opportunity to revise our work for further consideration at PLOS Genetics.

We have addressed all the comments raised during the second review process and have submitted a revised version of the manuscript along with the required documents, including the detailed response to reviewers, the marked-up manuscript with tracked changes, and the clean version of the revised manuscript. In the revised documents, including figures, to fit with the current nomenclature we had to change the labelling of the former Dp(16)9Yah to Dp(16)14Yah to avoid confusion with the Dp(11)9Yah already published.

Thank you again for your time and support. We look forward to hearing from you soon.

Kind regards,

Yann Herault

**Journal Requirements:**

1) Please provide an Author Summary. This should appear in your manuscript between the Abstract (if applicable) and the Introduction, and should be 150-200 words long. The aim should be to make your findings accessible to a wide audience that includes both scientists and non-scientists. Sample summaries can be found on our website under Submission Guidelines:
<https://journals.plos.org/plosgenetics/s/submission-guidelines#loc-parts-of-a-submission>

YH> done in the main manuscript page 2:

# Author summary

An extra copy of chromosome 21 causes Down syndrome (DS) and leads to intellectual disability and distinct facial features. To understand which genes drive these traits, we have engineered mouse models that mimic the DS genetic changes. By studying these mice, we identified regions of mouse chromosome 16, equivalent to human chromosome 21, that are associated with the head and face differences in Down syndrome (DS). We pinpointed two genes, *Dyrk1a* and *Ripply3*, whose increased activity disrupts normal facial development by altering the growth of specific cells during embryonic development. Notably, reducing Ripply3 activity in these mice corrected some facial abnormalities, highlighting its crucial role. These findings clarify how extra genetic material leads to DS features and suggest new therapeutic targets. The study also highlights the importance of animal models in elucidating the genetic and developmental basis of human conditions, such as DS. Additionally, the mechanism involving *Ripply3* connects DS with DiGeorge syndrome, another disorder with similar facial changes, suggesting shared pathways behind some features of both conditions.

2) We have noticed that you have uploaded Supporting Information files, but you have not included a list of legends. Please add a full list of legends for your Supporting Information files after the references list.

YH> The legends of the supplementary figures were previously in the Supporting Information file. We now moved them at the end of the main manuscript page 36 and removed them from the SI file

3) Please amend your detailed Financial Disclosure statement. This is published with the article. It must therefore be completed in full sentences and contain the exact wording you wish to be published.

1) State the initials, alongside each funding source, of each author to receive each grant. For example: "This work was supported by the National Institutes of Health (####### to AM; ###### to CJ) and the National Science Foundation (###### to AM)."
2) State what role the funders took in the study. If the funders had no role in your study, please state: "The funders had no role in study design, data collection and analysis, decision to publish, or preparation of the manuscript."
3) If any authors received a salary from any of your funders, please state which authors and which funders..
If you did not receive any funding for this study, please simply state: u201cThe authors received no specific funding for this work.u201d

4)  Please ensure that the funders and grant numbers match between the Financial Disclosure field and the Funding Information tab in your submission form. Note that the funders must be provided in the same order in both places as well.

YH>Considering the recommendations, the Financial disclosure statement should be as follow:

This work benefited from support provided by the Interdisciplinary Thematic Institute IMCBio, as part of the ITI 2021–2028 program of the University of Strasbourg, CNRS, and Inserm. It was funded through the French Investments for the Future Program under the following grants: IdEx Unistra (ANR-10-IDEX-0002), SFRI-STRAT’US (ANR-20-SFRI-0012), INBS PHENOMIN (ANR-10-INBS-07), and EUR IMCBio (ANR-17-EURE-0023).

Y.H. received funding from the European Union’s Horizon 2020 research and innovation program under grant agreement No. 848077 (GO-DS21). J.T.A. was supported by the National Agency for Research and Development (ANID), through the Scholarship Program DOCTORADO BECAS CHILE/2020 (Grant No. 72210028).

The funders had no role in study design, data collection and analysis, decision to publish, or preparation of the manuscript

1) Thank you for including an Ethics Statement for your study. Please include:

i) The full name(s) of the Institutional Review Board(s) or Ethics Committee(s).

**Figure resubmission:**

While revising your submission, please upload your figure files to the Preflight Analysis and Conversion Engine (PACE) digital diagnostic tool, <https://pacev2.apexcovantage.com/>. PACE helps ensure that figures meet PLOS requirements. To use PACE, you must first register as a user. Registration is free. Then, login and navigate to the UPLOAD tab, where you will find detailed instructions on how to use the tool. If you encounter any issues or have any questions when using PACE, please email PLOS at [figures@plos.org](mailto:figures@plos.org). Please note that Supporting Information files do not need this step. If there are other versions of figure files still present in your submission file inventory at resubmission, please replace them with the PACE-processed versions.

**Reproducibility:**

To enhance the reproducibility of your results, we recommend that authors deposit laboratory protocols in protocols.io, where a protocol can be assigned its own identifier (DOI) such that it can be cited independently in the future. Additionally, PLOS ONE offers an option to publish peer-reviewed clinical study protocols. Read more information on sharing protocols at <https://plos.org/protocols?utm_medium=editorial-email&utm_source=authorletters&utm_campaign=protocols>

*In compliance with data protection regulations, you may request that we remove your personal registration details at any time.* [*(Remove my information/details)*](https://track.editorialmanager.com/CL0/https:%2F%2Fwww.editorialmanager.com%2Fpgenetics%2Flogin.asp%3Fa=r/1/010f01981937834f-353dd86d-06fa-4b77-8343-889a72553169-000000/xMXQTIJ5O09XsdyLiGwIfZUEnpEOsCvn9Erq4kLKvj0=220)*. Please contact the publication office if you have any questions.*
